# The Emerging Functions of Long Noncoding RNA in Immune Cells: Autoimmune Diseases

**DOI:** 10.1155/2015/848790

**Published:** 2015-05-18

**Authors:** Keshav Raj Sigdel, Ao Cheng, Yin Wang, Lihua Duan, YanLin Zhang

**Affiliations:** ^1^Department of Nephrology, The First Affiliated Hospital of Xiamen University, Xiamen University, Xiamen 361003, China; ^2^Department of Nephrology, The First Hospital of Xiamen, Fujian Medical University, Xiamen 361003, China; ^3^Department of Rheumatology and Clinical Immunology, The First Affiliated Hospital of Xiamen University, Xiamen 361003, China

## Abstract

The long noncoding RNAs (lncRNAs) are RNA transcripts more than 200 nucleotides in length, which do not encode proteins. The lncRNAs are emerging as an important regulator of biological process, such as chromatin remodeling, gene transcription, protein transport, and trafficking through diverse mechanisms. The lncRNAs play crucial role in various multigenetics human diseases including cancers and neurological diseases and currently its role in autoimmune diseases is attracting many researchers. Recent studies have reported that differentiation and activation of immune cells, T cells, B cells, macrophages, and NK cells have correlation with lncRNAs, which have also an essential role in autoimmune diseases such as rheumatoid arthritis and SLE. Therefore, elucidation of the roles of lncRNAs in autoimmunity could be beneficial to understand the pathogenesis of autoimmune diseases. In this review article we attempt to highlight the recent progress regarding lncRNAs studies and summarize its role in autoimmune diseases.

## 1. Introduction

Among the 20,000 protein coding genes, only less than 2% of total human genome sequence has been reported [1]. Not surprisingly, at least 90% of the genome is actively transcribed into noncoding RNAs (ncRNAs), which have no protein coding potentiality [2]. A heterogeneous, novel class of long noncoding RNAs (lncRNAs) with length longer than 200 nucleotides is generally characterized as nonprotein transcript [3]. Over the past decade, with the speedy progress in high-throughput genetic sequencing technology more than 18,000 transcripts are annotated as lncRNAs that have been recognized in mammalian transcriptomes [4–6]. Based on it, various studies have revealed that lncRNAs are believed to form a major proportion of novel transcripts and known to be involved in number of functionally distinct biological and physiological processes including chromatin remodeling, gene transcription, RNA splicing, and protein transport diverse mechanisms [7, 8] and directly linked to human diseases including various cancers [9], Alzheimer's disease [10], and coronary artery disease [11]. Recently some studies have claimed that lncRNAs have certain roles in the different kinds of protein-coding and noncoding immune genes and their role in autoimmune diseases [12]. Furthermore, the lncRNAs act as a key regulator of inflammatory gene expression by a collaboration involving signal-dependent activation of transcription factors, transcriptional coregulators, and chromatin-modifying factors [13]. Nonetheless, to the date the exact mechanisms of lncRNA functions in autoimmunity are not well constituted. Here, we are going to review the lncRNAs functions associated with T cells, B cells, macrophages, and NK cells (Table 1), to the autoimmune diseases mainly in SLE, RA, and psoriasis (Table 2).

## 2. Classification and Characterization of LncRNAs

**Figure 1:**
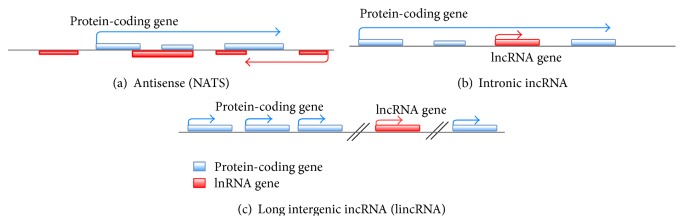
Classification and characterization of lncRNAs are based on their genomic localization with respect to the neighboring protein-coding gene. It is classified as overlapping lncRNAs including (a) natural antisense transcripts, or (b) intronic LncRNA and non-overlapping, (c) intergenic lncRNA; (lincRNAs), are transcribed from regions far away from protein-coding genes. The direction of arrow represents the different forms of transcription. Antisense lncRNA contains section of complementary sequences with the mature, spliced mRNA of the overlapping protein-coding gene. Intronic lncRNAs are transcribed within the intron of a protein-coding gene and therefore do not contain sequences complementary to the mature, spliced mRNA of the protein-coding gene [32].

Long noncoding RNA is a new class of transcripts which has been found to be pervasively transcribed in the genome, mutations, and dysregulations of lncRNAs that lead to diverse human diseases [30]. LncRNAs are classified on the basis of their genomic proximity to protein- coding genes as (1) sense or (2) antisense, when overlapping one or more exons for another transcript on the same or opposite strand, respectively, (3) bidirectional, when the expression of it and neighbouring coding transcript on the opposite strand is initiated in close genomic proximity, (4) intronic, a sequence which is derived entirely from within an intron of another transcript, and (5) intergenic, when it lies as independent unit within the genomic interval between two genes [7, 31]. Furthermore, at least three different groups can be categorized, namely, natural antisense transcripts (NATS), intronic RNA (IncRNAs), and long intergenic (intervening) noncoding RNA (lincRNAs) (Figure 1) [32]. These noncoding transcripts are often displayed as minimum or partial overlap with the coding sequence of the corresponding mRNAs regardless of protein coding potential but they may have an intrinsic function as mRNAs. Therefore, LncRNAs comprise a diverse class of transcripts that structurally resemble mRNAs but do not encode protein.

A study in human cell lines suggests that about 30% of lncRNAs are specifically expressed in the nucleus [33]. Several of them are involved in chromatin remodeling complexes and mediate genomic silencing [34]. Interestingly, the LncRNAs regulates the gene expression by interacting with its partner DNA, RNA and protein, which directly impacts upon human disease through various mechanisms, involved in epigenetic silencing, splicing regulation, translation control, regulating the apoptosis and cell cycle control. Every step of life cycle of gene from transcription to mRNA splicing and translations can be influenced by LncRNAs. However, lncRNAs might achieve regulatory specificity through modularity, collecting diverse combination of proteins, and possibly RNA and DNA interactions [35]. It also acts as evolutionary preserved transcripts of noncoding DNA succession, which have been implicated in the regulation of cellular differentiation [36] to genome rearrangement and inactivation of major tumor suppressor genes [32]. LncRNAs have been characterized to regulate the abundance of genomically neighbouring (*cis*-acting) or distal (*trans*-acting) gene products that were classified as* cis*-acting lncRNA-transcription dependent or* trans*-acting lncRNA-transcription dependent [37]. Moreover, the variety of molecular mechanism and biological roles of lncRNAs [38] are attributed but still limited to provide definitive understanding of its mechanisms. Recently, Marques and Ponting have described that most of* cis*-acting lncRNA to trait variation are low and* trans*-acting lncRNAs may be higher although it was difficult to establish until the full set of their genome-wide targets in their trait appropriate cells and tissue [39]. Taken together, paradigms of lncRNAs are gene imprinting Xist [6], Tsix [40], and air [41]; chromatin modification, HOTAIR [42]; RNA processing, MALAT-1 [43]; cell apoptosis and cell cycle control, Gas5, lincRNAs-p21 [34, 44]; and so forth. Shortly, it was estimated that more than 8000 lncRNA exist in the human organism [32]. Therefore, deregulated reflection of lncRNA is associated with a variety of multigenetic human diseases ranging from different organs cancers [12] to noncancer such as Alzheimer disease (BACE1-AS) [10], coronary heart disease (ANRIL) [11], myocardial infarction (MIAT) [45], and membranous nephropathy Xist [46] and to some alternation of immune system both in innate and adaptive [13, 47] which may lead to better understanding of infectious and inflammatory diseases.

## 3. LncRNA in Immune Cells and Its Role in Autoimmunity

**Figure 2:**
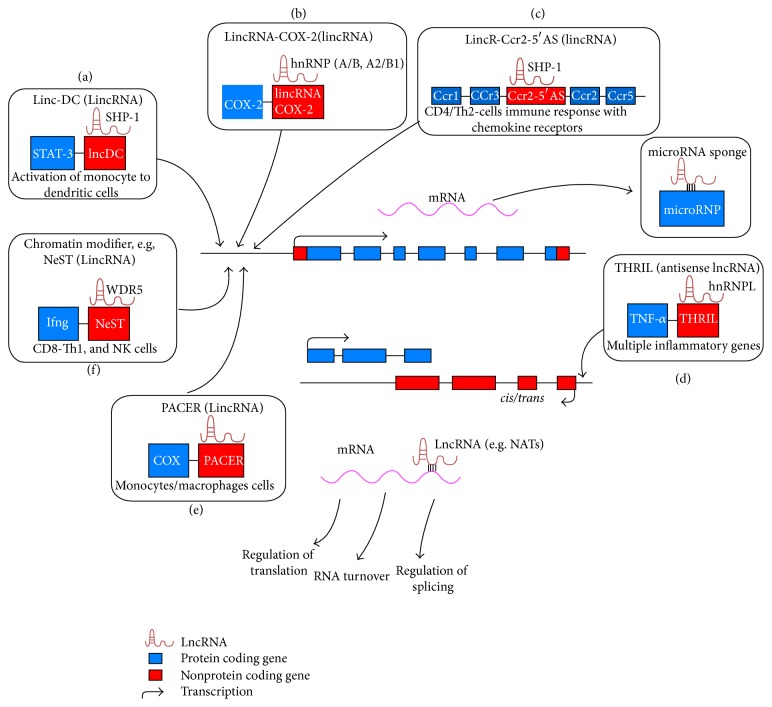
The functions of lncRNAs in autoimmune cells, T cells, B cells, macrophages, NK, and dendritic cells. (a) Lnc-DC expression is needed for differentiation of human monocytes into dendritic cells. Lnc-DC promotes STAT3 phosphorylation through inhibiting the action of Src homology region 2 domain-containing phosphatase-1 (SHP-1) [18]. (b) LincRNA-COX-2 is located in COX2 gene in mouse bone marrow-derived macrophages. It has extensive effects on inflammatory gene expression, repressing the transcription of anti-inflammatory genes in nonstimulated cells and promoting the expression of proinflammatory genes following Pam3Csk4 exposure via an interaction with hnRNP-A/B and A2/B1 [13]. (c) LincR-Ccr2-5′AS positively regulates the expression of genes involved in immunity particularly; lincR-Ccr2-5′AS regulates the transcription of several chemokine receptor genes in mouse, CD4+ TH2 cells, STAT-6 pathway [17]; (d) LincRNA (THRIL), as a key player in regulating the TNF-*α* and its expression was obviously lower during the acute phase of immune response. It was identified as an antisense lncRNA through a RNA-protein complex with hnRNPL and promotes TNF transcription [24]. (e) PACER is located upstream of the Cox2 transcriptional start site and is expressed in the antisense direction for innate immune response helping in gene expression, production of inflammatory mediators, and finally differentiation of monocytes/macrophages and dendritic cells [25]. (f) Chromatin modifier, for example, NeST, a lincRNA located downstream of Ifng which promotes the transcription of Ifng, WDR5, core submits of the MLL H3K4 methyltransferases, and facilitate the histone methylation at the Ifng locus, which promotes the transcription of Ifng in Th1 CD4+/CD8+ T cells. lncRNA also typically interacts with other transcripts and regulates miRNAs pathway, translation splicing, and RNA turnover [15]. LncRNAs characterized to regulate the abundance of genomically neighbouring (*cis- and trans-*acting) gene products [37].

Development of autoimmune disease is associated with epigenetic mechanism that modulates the gene networks in response to complex profiles of environment [48]. The differentiation and activation of immune cells are dependent on synchronized set of transcriptional and posttranscriptional events. Chromatin-modifying complexes admeasure the regions of the genome which are accessible to transcription factors and regulate the transcription of immune genes [49, 50]. Importantly, the essentiality of micro-RNAs for normal immune functions, immune cell development, and prevention of autoimmune diseases had been studied [51, 52]. However, LncRNA study is emerging as important regulators of immune cells differentiation and activation in recent years [47]. Here, we are going to summarize the functions of long noncoding RNAs associated with immune cells (Figure 2).

### 3.1. Long Noncoding RNA in T Cells

The study of LncRNAs function in the immune system found them as important regulators of the various biological processes recently. Th1 helper cells are crucial for organizing for adaptive immune responses to variety of pathogens; they are involved in various pathogeneses of different types of immunological diseases including autoimmune diseases, allergy, and asthma [53]. LincRNAs, TMEVPG1 (also termed, LincR-*Ifng*-3′AS), recognized in human and mouse CD8+ T cells, has been displayed to be located within a cluster of cytokines genes, controlled Theiler's virus load in infection of CNS [54]. Th1 cells specific and selective transcription factor T-bet/Stat with TMEVPG1 controls the expression of interferon gamma (IFN-*γ*) [14]. Gomez et al. [15] described lncRNAs, also called NeST, which interact with WDR5, a core submits of the MLL H3K4 methyltransferases, and facilitate the histone methylation at the* Ifng* locus in CD8+/Th1 cells. Genome-wide expression analyses revealed that presence of hundreds of lncRNAs in CD8+ T cells from human and mouse spleen by using custom array suggests an essential role of lncRNAs in the differentiation and activation of lymphocytes [16]. Hu et al. [17] have performed an experiment in RNA-Seq of 42 subsets of thymocytes and mature peripheral T cells and identified 1,524 genomic regions that generate lincRNAs; key transcription factors including T-bet and STAT4 for the CD8+ and GATA-3 and STAT6 for the CD4+ lineages were largely accountable for the lineage-specific expression of T cell lincRNAs (LincR-Ccr2-5′AS), for the better understanding of lncRNAs in the development and differentiation of T cells. Currently, a novel study has appeared in the immunology; STAT3- binding lncRNAs lnc-DC, which is exclusively expressed in human conventional dendritic cells, bound directly to STAT3 signaling molecule in the cytoplasm, suggested that lncRNAs can affect cellular differentiation (monocyte into dendritic cells identified, lnc-DC) and function by directly interacting with signaling molecules in the cytoplasm and upregulate their posttranslational modification [18]. Interestingly, Lnc-DC, a specific regulator of DC differentiation and function, may have potential role to clinical diseases involving DC dysfunction and may have influence for the activation of CD4+ T cells response. In another study, LncRNA, small antisense transcript of ZFAT gene expression, has been equally detected in CD4+ T cells, CD8+ T cells, CD19+ B cells, and CD14+ monocytes in autoimmune thyroid disease (AITD) [19]. Mycosis fungoides (MF) and Sezary syndrome are common form of cutaneous T cell lymphoma (CTCL); Sezary cell associated to LncRNAs (SeCATs) has been identified in a broad spectrum of normal human [20].

### 3.2. Long Noncoding RNA in B Cells

B lymphocytes are formed in human bone marrow. Principle functions of B cells are to make antibodies against antigen, to perform the role of APCs, and to develop into memory cells after activation by antigen interaction [55]. Interestingly, Gomez et al. [15] have demonstrated that the lncRNAs can regulate the immune response in animal model of infection with the help of T cells. In comparison to T cells, very little knowledge of B cells functions of lncRNAs is known; so far Bolland et al. [56] have explained a role of lncRNAs in the chromatin remodeling associated with the variable, diversity, and joining (V/D/J) genes recombination required to produce antigen receptors (Ig or TCR). Additionally, another study has shown that transcription of these antisense and sense lncRNAs is linked to looping of VH regions into close proximity with DJH region during recombination of pro-B cells; consequently it has been identified as the full transcriptome of sense and antisense transcripts throughout the Igh locus [21]. These processes occurred within transcription factors but the mechanism has not been broadly defined yet. A current study has revealed that profiling of lncRNAs expression during differentiation of monocyte into dendritic cells (Mo-DC) identified lnc-DC, which was exclusively upregulated [18]. Furthermore, this study showed significant characteristics of lnc-DC gene loci, which revealed that low or absent Lnc-DC expression in human blood CD20+ cells, B cells; CD14+, monocytes; CD34 cells, mobilized hematopoietic progenitor cells in human and H1-hESC (human embryonic stem cells) in periphery blood. Thus, lnc-DC is more exclusively expressed in human cDC (conventional dendritic cells) of the hematopoietic system than other DC markers. Functionally, Lnc-DC knockdown impacted protein coding genes, resulting in an antigen uptake, impaired allogenic CD4+ T cell proliferation, and reduced the strength of cytokine release. In LncRNAs, SAS-ZFAT gene was exclusively and specifically expressed in CD19+ B cells in peripheral blood lymphocytes and could have crucial roles in B cell function and determines the etiology of AITD [19].

### 3.3. Long Noncoding RNA in Macrophages

It has already been cleared through various studies that dead and dying cells in healthy persons are removed by macrophages in an anti-inflammatory environment. As antigen presenting cells, macrophages have an additional role optimizing the function of both the innate and acquired immune response [57]. The recognition and function of lncRNAs in monocytes/macrophages have not been studied abundantly. Not surprisingly, macrophages express the highest basal level of ptprj/CD148 (a tyrosine phosphatase that has tumor suppressor-like activity); its level gets changed by the treatments of LPS, TLR, and CSF-1 in different models. In this study they identified a 1,006 nucleotide long noncoding RNA species, ptprj-as1, that is transcribed antisense to ptprj. Transcribed ptprj-as1 is significantly expressed in macrophage enriched tissue, which was transiently induced by TLR ligands parallel to ptprj [22]. Thus, ptprj coding transcript may lead to modulation of inflammation directly linked with microphages. Myeloid (mDCs), CD11c+ dendritic cells (DCs, Antigen Presenting Cells) express lncRNAs-*COX2(Ptgs2)* when they are stimulated with lipopolysaccharide, an activator of TLR4 signaling through NFkB [23]. Similarly, Li et al. [24] have analyzed the change in expression of lincRNAs upon activation of innate immune signaling in THP1 macrophages and identified an unannotated LincRNAs, termed THRIL, as a key player in regulating the TNF-*α* and its expression was obviously lower during the acute phase of Kawasaki disease. LincRNA has shown that functions through a RNA-protein complex with hnRNPL [44] have critical role as regulator of physiological and pathological inflammatory immune responses. Many lncRNAs regulate transcription with hnRNPs (multifunctional nuclear RNA), have been identified as specific binding partners for lincRNA-cox2 in macrophages in both nuclear and cytoplasm, and are a major regulator of immune genes [13]. Likewise, LncRNAs PCAER, as a new potential target for Cox-2 modulation in inflammation and cancer, mediate Cox2 expression in human monocytes, as its direct upstream of the Cox2 transcriptional start side and expressed in the antisense directions. By this phenomenon widespread change in lncRNAs expression following the activation of innate immune response helps in gene expression, production of inflammatory mediators, and finally differentiation of monocytes into macrophages and dendritic cells [25].

### 3.4. Long Noncoding RNA in Natural Killer Cells

NK cells are a type of cytotoxic lymphocyte which is important to the innate immune system. The cytolytic activity of NK cells is modulated by the presence or absence of class I MHC molecules on target cells. NK cells use cell-surface receptors for class I MHC to assess the condition of target cells [58]. KIR (killer cell immunoglobulin-like receptor) is the major type of class I receptor expressed by NK cells. Wright et al. [26] reported the presence of an intron 2 promoter in several KIR genes that produce a spliced antisense transcript LncRNAs. The KIR antisense lncRNA is detected in progenitor cell lines and its overexpression in NK cells leads to decreased expression of KIR protein coding genes. KIR antisense lncRNA overlaps with KIR-coding exons 1 and 2, as well as the proximal promoter that is upstream of KIR. Transcription of KIR antisense lncRNA seems to be regulated by myeloid zinc finger one (MZF-1) that leads to silencing of KIR through an anonymous mechanism.

## 4. Long Noncoding RNA and Autoimmune Diseases

LncRNAs are coming into existence as new hopes in the autoimmune diseases paradigm demonstrating their potential roles in innate and adaptive immune system, which may be crucial players of autoimmunity. Here are few autoimmune diseases which have some association with lncRNAs expression though its role is not well established in the literature.

### 4.1. LncRNA in Systemic Lupus Erythematosus (SLE)

Systemic lupus erythematosus (SLE) is a prototypic systemic autoimmune disease which involves a complicated interaction between the innate and the adaptive immune system, loss of immunological tolerance to self-nuclear antigen, and antibody production [59]. The production of autoantibodies targeting double stranded DNA (dsDNA) and other nuclear autoantigens is the main characteristic of this disease [48]. Despite the huge numbers of research, the etiology of SLE remains subtle and it is thought that genetic and epigenetic predisposition joined with familiar and unfamiliar environmental factors play pivotal role in the development of SLE [60]. T cells, B cells, and dendritic cells are critical cells for SLE pathogenesis [61, 62]. Not surprisingly, the cellular and molecular role of small noncoding RNAs (miR-21, miR25, miR125, miR146a, and miR186, etc.) in the regulation and pathogenesis of SLE have been reported [52]. However, lncRNAs cellular and molecular mechanisms are still unexplored. Recently, Shi et al. [27] in their small cohort studies have compared the whole transcriptome analysis of purified monocytes from 9 female patients with SLE to the gene expression of 8 healthy controls; while doing so they found SLE specific alternative splicing, alternative polyadenylation, and novel loci transcription, an effect replicated by LPS treatment of control monocyte. In addition, the study identified the decrease expression of noncoding RNAs in Aicardi Goutieres syndrome, an infantile-onset disorder with features of lupus, in murine model to drive type I interferon. Pri-miRNA was clearly induced in SLE patients. These small noncoding RNAs are processed to suppress the translation and regulated the several messenger RNAs. Two specific pri-miRNAs were significantly upregulated in SLE monocyte compared with healthy controls, whereas two miRNAs showed decreased message levels, which shows that the pri-miRNA levels in SLE monocytes are functionally pertinent. Additionally, LncRNAs are less likely to get changed in SLE compared to other RNA classes, while the locations of some significantly changed lncRNA have suggested their involvement in SLE. For example, both* HIVEP2* itself and a lncRNAs about (800–1500 base) upstream of its TSS were significantly upregulated in SLE. Interestingly, LncRNAs which were located on chromosome 6q25.3 had commonly dysregulated in SLE monocytes. Moreover, protein coding genes TAGP, SOD2, WTAP, and ACAT2 transcription levels were all upregulated in SLE, while the other coding gene, FNDC1 transcription level, was low and downregulated in SLE.

### 4.2. LncRNA in Rheumatoid Arthritis (RA)

Rheumatoid arthritis is a systemic autoimmune disorder characterized by chronic inflammation of synovial tissue that results in irreversible destruction of small to medium size joints [63]. The major cellular contributors in RA are T and B lymphocytes, neutrophils, macrophages, and proliferating fibroblast-like cells. Alteration of the synovial microenvironment by proinflammatory cytokines and chemokines attracts T, B, and APCs cells and encourages secretion of protease that promotes joint destruction [64]. Most of the studies in cultured cells and mammals have proved that micro-RNAs (miRNAs) play a critical role in the pathogenesis of RA (miR-124a, miR-146a, and miR-155) [51]. In human, within the past few years molecular studies exhibited a functional role of lncRNA in the cancer development. However, lncRNAs role in autoimmune disease like in RA is not well established. Recently, Müller et al. [28] have investigated ten patients, who were suffering from RA, and applied two biological treatments adalimumab (anti-TNF-*α*) and tocilizumab (anti-IL-6R); in the mean time they measured the serum level of cytokines TNF-*α* and IL-6, respectively, where they found that lincRNA (used total number, 7.419 lincRNA) has been regulated by TNF-*α* and superior to IL-6 in CD14 monocytes in vivo in human subjects with rheumatoid arthritis. These cytokines have a specific correlation with lincRNA transcription. Therefore, the interregulation of lincRNA may be important intracellular molecular effectors of different cytokines in cells of innate immune system in human in vivo in the context of rheumatoid arthritis.

### 4.3. LncRNA in Psoriasis

It is a hyperproliferative inflammatory skin diseases, PRINS (psoriasis—associated RNA induced by stress), a lncRNA that harbour two ALu elements, which is upregulated in the skin of patients with psoriasis, which contributes to psoriasis via the downregulation of G1P3, a gene coding protein with antiapoptotic effects in keratinocytes [29].* In silico* structural homology studies have recommended that PRINS act as a noncoding RNA. PRINS is transcribed by RNA polymerase II and is expressed at different levels in various human tissues. Real time reverse transcription-PCR analysis exhibited that PRINS has higher expression in the uninvolved epidermis of psoriatic patients than in both psoriatic lesion and healthy epidermis, suggesting that PRINS has a role in psoriatic susceptibility. Furthermore, downregulating the RNA level of PRINS by RNA interference can impair cell viability after serum starvation but not under normal serum conditions. It was ascertained that PRINS may also function as a “riboregulator” to govern the expression of other genes involved in the proliferation and survival of cells revealed to stress.

### 4.4. LncRNA with Other Autoimmune Diseases

#### 4.4.1. Autoimmune Thyroid Disease

Autoimmune thyroid disease (AITD) is caused by an immune response to self-thyroid antigens and has a significant genetic component. It includes Graves disease (GD) and Hashimoto's thyroiditis (HT) [65]. LncRNAs play crucial role in AITD. A study has revealed the importance of genetic component in AITD. Shirasawa et al. enrolled 515 affected individuals and 526 controls in a study and found the correlation of T-allele of SNP Ex9b-SNP10 to the increased risk for autoimmune thyroid disease [19]. The EX9b-SNP10 dwells in intron 9 of protein coding gene ZAFT (zinc-finger gene in ATD) and in the promoter region of LncRNA, SAS-ZFAT, which is the small antisense transcript of ZAFT gene. Being with the SNP (EX9b-SNP10), SAS-ZFAT expression gets exclusively upregulated, while it decreases the expression level of truncated-ZAFT (TR-ZFAT).

#### 4.4.2. Sjögren's Syndrome (SS)

Sjögren's syndrome is an autoimmune disease characterized by inflammation of exocrine glands mainly salivary and lachrymal glands, leading to dry mouth and dry eye symptoms [66]. Typically age of onset is 40–50 and there is female predominance. The hallmark of the SS is B cell hyperactivity that is revealed by hypergammaglobulinemia, circulating immune complexes, and anti-Ro/SSA and anti-La/SSB autoantibodies [67]. A study of MSG (minor salivary gland) RNA samples, primary SS yielded 94 bp fragments of coxsackievirus B4 (CVB4) p2A genes, which may elucidate the possible role of CVB4 in induction and maintenance of primary Sjögren's syndrome [68]. However, in Pubmed and MeSH database the study of lncRNA in Sjögren's syndrome is not accessible yet.

## 5. Conclusion

In a nutshell, the LncRNAs are RNA transcript more than 200 nucleotides in length, which do not encode proteins and play a crucial role in autoimmune diseases such as SLE, RA, and psoriasis AITD, including various multigenetic human diseases. Activation, differentiation, and imbalance expression of immune cells, T cells, B cells, macrophages, and NK cells alter the autoimmunity which may have direct link to lncRNAs. However, the identification of lncRNAs expression in autoimmune diseases is largely unexplored. Majority of transcribed DNA encode noncoding RNAs. The relative proportion of noncoding genomic DNAs increases the developmental complexity which signifies that ncRNAs may serve more critical biological functions in autoimmune diseases that may resemble different organ cancers. A better understanding of long noncoding RNAs is emerging as key regulators of diverse biological process especially by immune cells and the molecular mechanism of autoimmunity. Based on aforementioned studies (Table 2) we can conclude that determining individual role of lncRNAs in autoimmune diseases remains a challenge, additional studies of lncRNAs are very essential for the better understanding of autoimmune diseases.

## Figures and Tables

**Table 1:** LncRNAs associated transcription factors, genes, and cells in autoimmunity.

	LncRNAs	Cells	References
	TEMVPG1 (LincR-Ifng-3′AS)	CD8+	[14]
	NeST (WDR5)	CD8+/CD4+	[15]
	Lef1as (Wnt)	CD8+	[16]
	Ptpre (Jak-Stat)	CD8+	[16]
	Il2ra	CD8+	[16]
T-cells	LincR-Ccr2-5′AS, (GATA-3)	CD4+/Th2	[17]
	LincR-Gng2-5 (STAT4)	CD8+/Th1	[17]
	LincREpas1-3′s (STAT6)	CD4+/Th2	[17]
	LincR-Ifng-3′AS (T-bet)	CD8+/Th1	[17]
	Lnc-DC (STAT3)	CD4+	[18]
	TR-ZAFT	CD4+, CD8+	[19]
	SeCATs	CD4+	[20]

B cells	Linc-DC	CD20+B cells,	[18]
	SAS-ZAFT	CD19+B cells	[19]
	Igh locus (DJH)	pro-B cells	[21]

	lincRNA-Cox2 (hnRNP-A/B, A2/B1)	Macrophages	[13]
	linc-DC	CD14 monocyte	[18]
Macrophages	ptprj	CD148	[22]
	lincRNA-Cox2 or Ptgs2 (NFkB)	TLR4, CD11c+dendritic cells	[23]
	THRIL (hnRNP-L)	THP1/Macrophages	[24]
	PACER	Monocytes/DC	[25]

NK cells	KIR	NK cells	[26]

**Table 2:** List of LncRNA studies, associated with autoimmune diseases.

LncRNA	Diseases name	References
SAS-ZFAT	Thyroid disease	[19]
THRIL	Kawasaki disease	[24]
FNDC1, TAGP, SOD2, WTAP and ACAT2	SLE	[27]
LincRNA (total number 7.419)	RA	[28]
PRINS	Psoriasis	[29]

## Abbreviations

Air: Antisense Igf2r
AITD: Autoimmune thyroid disease
ANRIL: Antisense noncoding RNA in the INK4 locus
BACE1-AS: *β*-site amyloid precursor protein- (APP-) cleaving enzyme
CNS: Central nervous system
Gas5: Growth-arrest-specific 5
HIVEP2: Human immunodeficiency virus type I enhancer binding protein-2
hnRNPL: Heterogeneous nuclear ribonucleoprotein L
HOTAIR: HOX antisense intergenic RNA
Ifng: Interferon gamma
KIR: Killer cell immunoglobulin-like receptor
lincRNA: Long intergenic noncoding RNA
lncRNA: Long noncoding RNA
lnc-DC: Long noncoding dendritic cells
LPS: Lipopolysaccharide
MALAT1: Metastasis-associated lung adenocarcinoma transcript 1
MIAT: Myocardial infarction-associated transcript
miRNA: microRNA
MLLH3k4: Mixed lineage leukemias histone H3 at lysine 4
NATs: Natural antisense transcripts
NeST: Nettoie Salmonella pas Theiler's
pri-miRNAs: Primary miRNAs
SLE: Systemic lupus erythematosus
STAT3: Signal transducers and activator of transcription 3
TLR: Toll-like receptor
THRIL: TNF-*α* and hnRNPL related immunoregulatory lincRNA
TSS: Transcription start site
XIST: X-inactive-specific transcript.
